# Reinforcement Learning With Vision-Proprioception Model for Robot Planar Pushing

**DOI:** 10.3389/fnbot.2022.829437

**Published:** 2022-03-02

**Authors:** Lin Cong, Hongzhuo Liang, Philipp Ruppel, Yunlei Shi, Michael Görner, Norman Hendrich, Jianwei Zhang

**Affiliations:** TAMS Group, Department of Informatics, Universität Hamburg, Hamburg, Germany

**Keywords:** reinforcement learning, robot manipulation, planar pushing, multimodal, Variational Autoencoder, Soft Actor-Critic

## Abstract

We propose a vision-proprioception model for planar object pushing, efficiently integrating all necessary information from the environment. A Variational Autoencoder (VAE) is used to extract compact representations from the task-relevant part of the image. With the real-time robot state obtained easily from the hardware system, we fuse the latent representations from the VAE and the robot end-effector position together as the state of a Markov Decision Process. We use Soft Actor-Critic to train the robot to push different objects from random initial poses to target positions in simulation. Hindsight Experience replay is applied during the training process to improve the sample efficiency. Experiments demonstrate that our algorithm achieves a pushing performance superior to a state-based baseline model that cannot be generalized to a different object and outperforms state-of-the-art policies which operate on raw image observations. At last, we verify that our trained model has a good generalization ability to unseen objects in the real world.

## 1. Introduction

**Figure 1:**
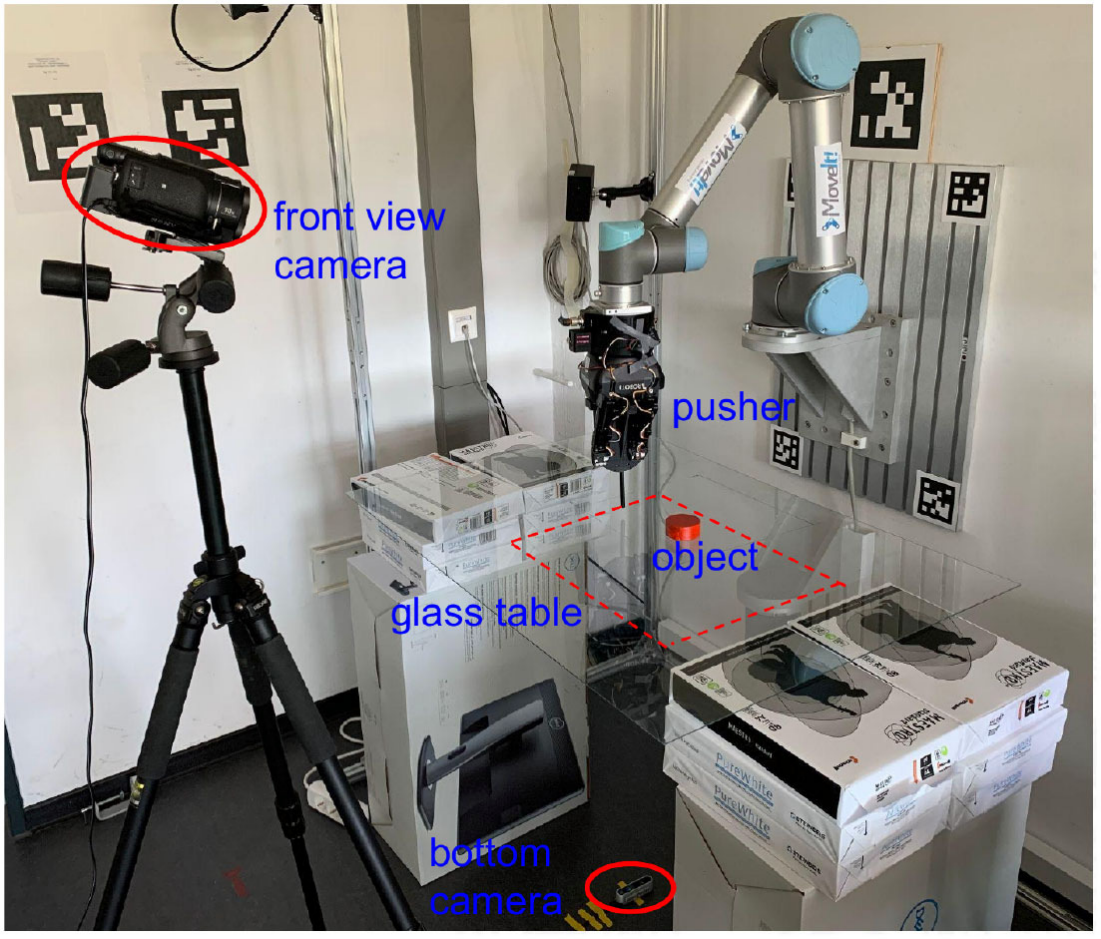
Our experiment platform consists of a UR5 robot with a Robotiq 3-finger gripper that grasps the 3D-printed vertical pusher rod. Its cylindrical part, designed to touch and push the moving object, is 6 mm in diameter. The pushing target placed on the transparent table is painted red to obtain masks easily through color filtering. The bottom camera (to get the input image) is set right below the table, and another front camera is added to record experimental videos.

**Figure 2:**
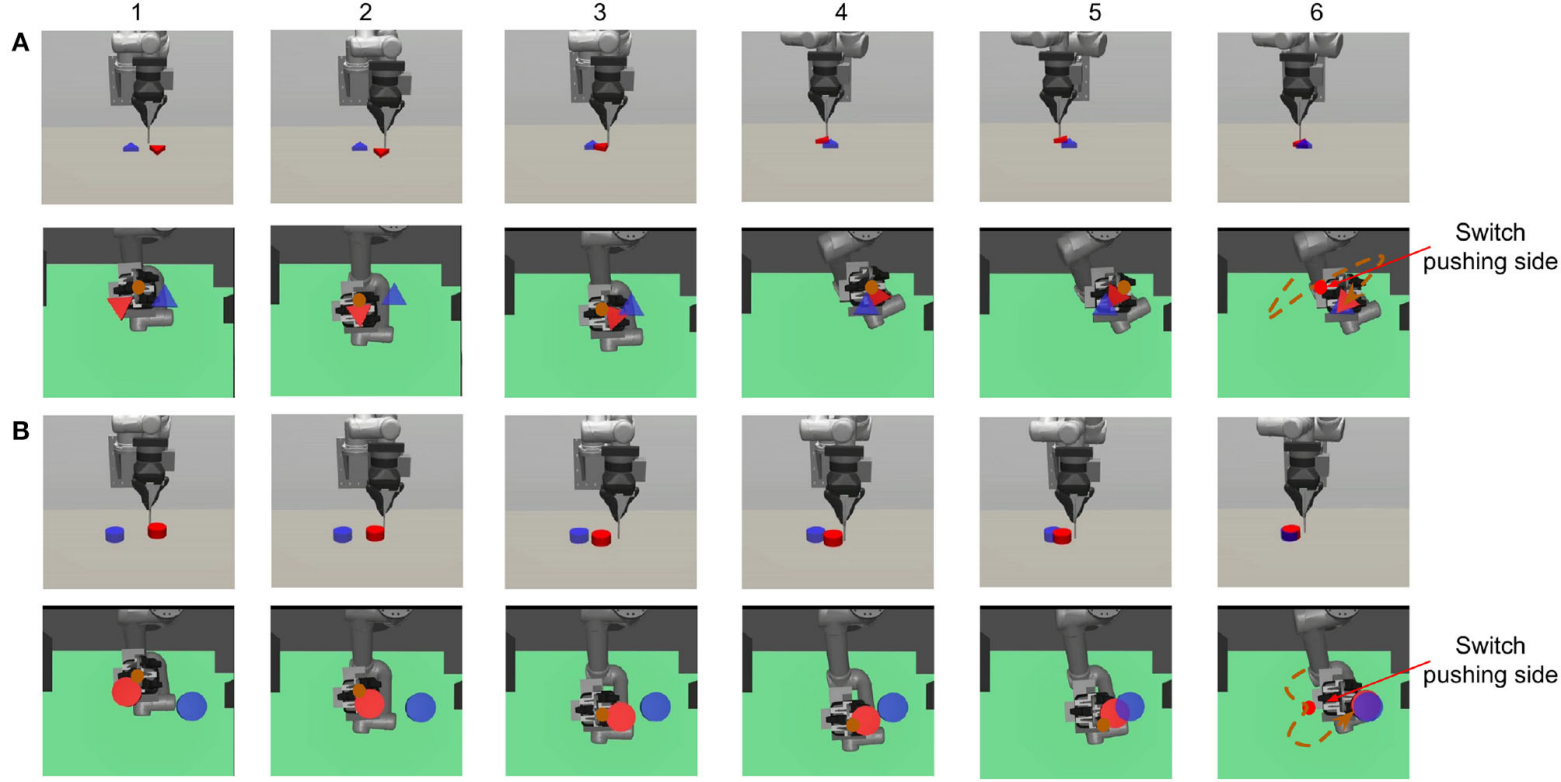
The figure shows two pushing processes: **(A)** triangle and **(B)** cylinder. Both the initial and target position are generated randomly at the beginning of each episode. We show both the front view (first row) to give an overall robot-object scene and the bottom view (second row) to show what the robot sees. The transparency of the table is set to 0.3 in the simulation. The brown point in (1–5) and the dashed line in (6) represent the pusher position and trajectory. In both **(A,B)**, we can see that the robot learns to first choose a proper initial pushing direction and to switch the pushing side when the object deviates from the target. The final orientation error in **(B)** is explained in Section 3.1.

Planar object pushing with a single contact is typical underactuated robot manipulation. Solutions to most robot manipulation problems can be divided into model-based or model-free methods. Our previous work (Cong et al., 2020) focused on a model-based method; we built a data-driven recurrent model which adapts to the real interaction dynamics after several pushing interactions using the proposed RMPPI algorithm as the controller. However, one limitation of the previous method is that the robot cannot effectively switch pushing sides according to the object's current pose during the pushing process. Another limitation is that we need AprilTag (Olson, 2011) to locate the object in real-time. In this work, we train a model-free reinforcement learning (RL) policy that takes the raw image and the pusher position as input (Figure 1). After enough training episodes in simulation, the trained agent learns to make good decisions on switching the pushing side both in simulation (Figure 2) and in the real world (**Figure 9**).

Extracting task-relevant information such as the object's shape and pose features from image observations is essential to enhance the robot's generalization ability to manipulate different objects. However, in most situations, the raw image from the camera always includes complicated components such as noisy background, which is hard for the robot to understand, and, therefore, it can hardly pay attention to the key components in the image. In this work, we extract useful information by segmenting an object mask from the image and constructing a latent representation through a Variation Autoencoder (VAE). As the decoder from the trained VAE can roughly reconstruct most of the original masks from the latent features, these features should include the object's pose and shape information. The robot state could be inferred from the camera image through a deep network, but this is not necessary. Unlike the object state, the robot state including the end-effector pose can be obtained directly from the hardware system. We, therefore, propose a vision-proprioception model to fuse the latent features and robot states as the RL inputs effectively. We train the agent to push objects to target positions using a carefully designed reward function (refer to Section 2.3.1). The models are evaluated by the distance between final positions and target positions.

With this paper, we contribute multiple insights:

- We introduce a VAE-based vision-proprioception model representing environment states for goal-conditioned RL.
- We apply our model in the RL to train the robot to learn planar object pushing.
- We train the model in simulation and do comparison experiments with models taking only raw images as inputs.
- We verify our model's ability to generalize with unseen objects on a real robot platform.

## 2. Materials and Methods

### 2.1. Related Work

#### 2.1.1. Planar Object Pushing

Planar object pushing is an active research topic in robot manipulation, the essence of which is the single contact underactuated control. Both model-based and model-free methods have been proposed to solve the problem. Building a data-driven model (Bauza and Rodriguez, 2017; Bauza et al., 2019) with large amounts of robot-object interaction datasets or an analytical model (Kloss et al., 2020) with specific physical meanings and then applying Model Predictive Control (MPC) as robot control strategy is the general model-based solution. However, collecting real interaction trajectories is very time-consuming, while physics parameters in real situations can only be approximated, making the model-based methods hard to apply. A model-free method like RL can be used to get actions directly from the ground truth state (Peng et al., 2018), or raw pixel images (Zadaianchuk et al., 2020). However, generalization of the model to different manipulation objects is hard for ground truth input, while extracting useful features such as object shape, size, and the robot's relative position efficiently from raw images for a subsequent policy network is always tricky.

#### 2.1.2. Vision-Based RL

Learning from pixel input is a challenging problem in RL. Convolutional neural networks (CNNs) (LeCun et al., 1998) are always used as an encoder in modern RL algorithms to get spatial features from images. Recent work achieves impressive results on DeepMind Control Suite, and OpenAI Gym benchmarks with learning tricks like image reconstruction (Ha and Schmidhuber, 2018; Yarats et al., 2019), data augmentation (Laskin et al., 2020), and contrastive learning (Laskin et al., 2020). However, low data sample efficiency during exploration makes it hard to train directly on real robot platforms. In our work, automatic domain randomization (ADR) (Akkaya et al., 2019) is applied to bridge the gap between simulation and the real world.

Reinforcement learning consistently achieves better learning performance given ground truth states compared with pixel inputs (Laskin et al., 2020). One reason is that an image input usually needs a more complicated network structure like CNNs to process spatial features, which increases training difficulty. Also, it is challenging to uncover attended regions and eliminate interference from unrelated pixel perturbations without any privileged information on the image. Top-down attention mechanisms are used to force the agent to focus on task-relevant information (Manchin et al., 2019; Mott et al., 2019). A partially observable ground truth state can also be used to train the agent with image input. For example, the asymmetric actor-critic method (Pinto et al., 2017) is used to improve both the robustness and sample efficiency *via* access to the real state while providing only images for the actor (Salter et al., 2020). A deep autoencoder is used to acquire a set of feature points from the image and perform the action planning with these feature points (Finn et al., 2016). However, a linear-Gaussian controller needs to be pre-trained as the exploration strategy, making the whole training process complex.

#### 2.1.3. Self-Supervised Feature Representation

In our case, task-relevant information includes both the object and target's shape, size, and pose, but no ground truth representation can be obtained directly. Learning rich representations from high dimensional pixel data for control is an active research area in robotics and RL. Using dense (pixelwise) visual description as the representation has been proven effective in visual correspondence estimation (Choy et al., 2016; Schmidt et al., 2016). A self-supervised system is proposed to learn consistent dense representation for robotic manipulation (Florence et al., 2018) while a compact state space is necessary for our goal-based RL task. Contrastive learning (Oord et al., 2018; He et al., 2020) is always used as an unsupervised learning approach to extract useful representations from high-dimensional data, which has been applied successfully to image recognition (Henaff, 2020) and RL (Anand et al., 2019; Laskin et al., 2020). Contrastive loss is also used in time contrastive networks (TCNs) (Sermanet et al., 2018), which learn state representation using temporal information from unlabeled demonstration videos. Other methods rely on generative reconstruction loss like VAE (Kingma and Welling, 2014) and its variations to compress images to latent vectors. The latent vector representing what the agent sees at each time frame is then fed into recurrent neural networks (RNNs) to predict the future (Ha and Schmidhuber, 2018). Motion skills can be performed directly in the latent space, which is mapped from camera images through a deep spatial autoencoder (Finn et al., 2016). Latent state models are also used in meta-RL to accelerate representation learning (Zhao et al., 2020). The state representation is learned from a sequence of visual observations in real-world robotic control tasks.

### 2.2. Theory

This section presents the prior theory used in our method in detail, including goal-conditioned RL, VAEs, and our mixed model for RL.

#### 2.2.1. Goal-Conditioned RL

**Figure 3:**
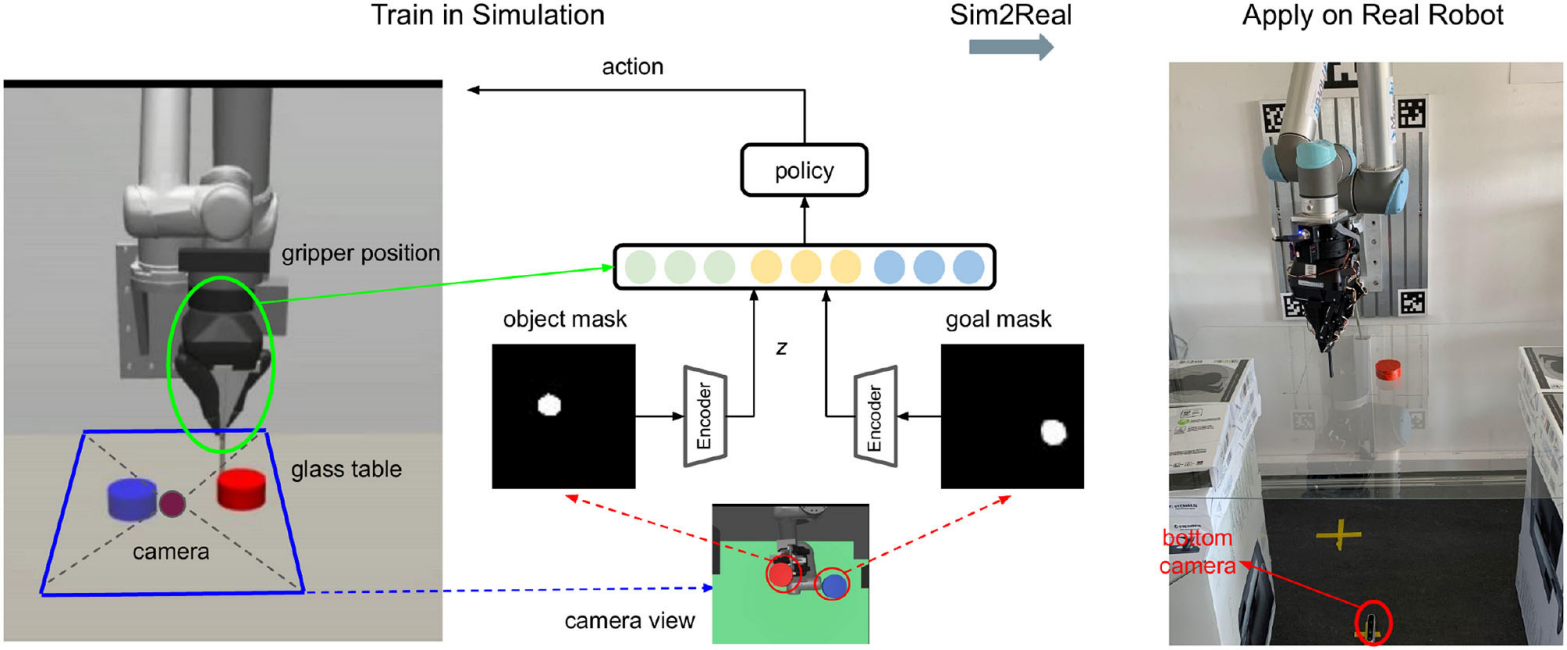
Overview: The training process in simulation is shown on the left, and the model is transferred directly to the real platform (on the right side). The camera is put below the transparent table to avoid possible occlusions between the camera view and the object during manipulation. Color filtering is applied to the image from the bottom camera to obtain both the object (red) and goal (blue) masks. The two masks are fed into the pre-trained autoencoder and then concatenated with the gripper position into a fusion space, where the policy does planning.

This task can be described briefly as pushing the object from a random initial pose to a given target position (represented by a mask image) like in Figure 3. We consider a finite-horizon discounted Markov Decision Process (MDP) defined by $\left(S,A,p,r_{g}\right)$, where $s_{t}\in S$ and $a_{t}\in A$ are continuous states and actions, respectively, *p*(*s*_*t*+1_|*s*_*t*_, *a*_*t*_) is the dynamics function, *r*_*g*_ is a function parametrized by the goal *g* as in the goal-conditioned RL (Schaul et al., 2015), computing the reward for reaching new state *s*_*t*+1_. Then, the task of the agent is to maximize the expected goal-conditioned return:


(1) $$\pi_{\phi }^{*}=\underset{\pi }{\text{argmax}}E_{g\in G}\left[E_{\tau (\pi )}[\sum_{t=0}^{T}\gamma^{t}r_{g}(s_{t})]\right]$$

by conditioning its policy π_ϕ_(*a*_*t*_|*s*_*t*_, *g*) on the goal *g*, where *G* is the distribution over the goal space during the training process. The goal space and state space play important roles during training as both Q-function *Q*_ψ_(*s*_*t*_, *a*_*t*_, *g*) and policy π_ϕ_(*a*_*t*_|*s*_*t*_, *g*) are computed within the two spaces. However, taking high-dimensional image observations as state and goal spaces makes the optimization hard (Nair et al., 2018). So in our work, we use low-dimensional and structured representations from a VAE for both of these. Related details are presented in Section 2.2.3.

#### 2.2.2. Variational Autoencoders

**Figure 4:**
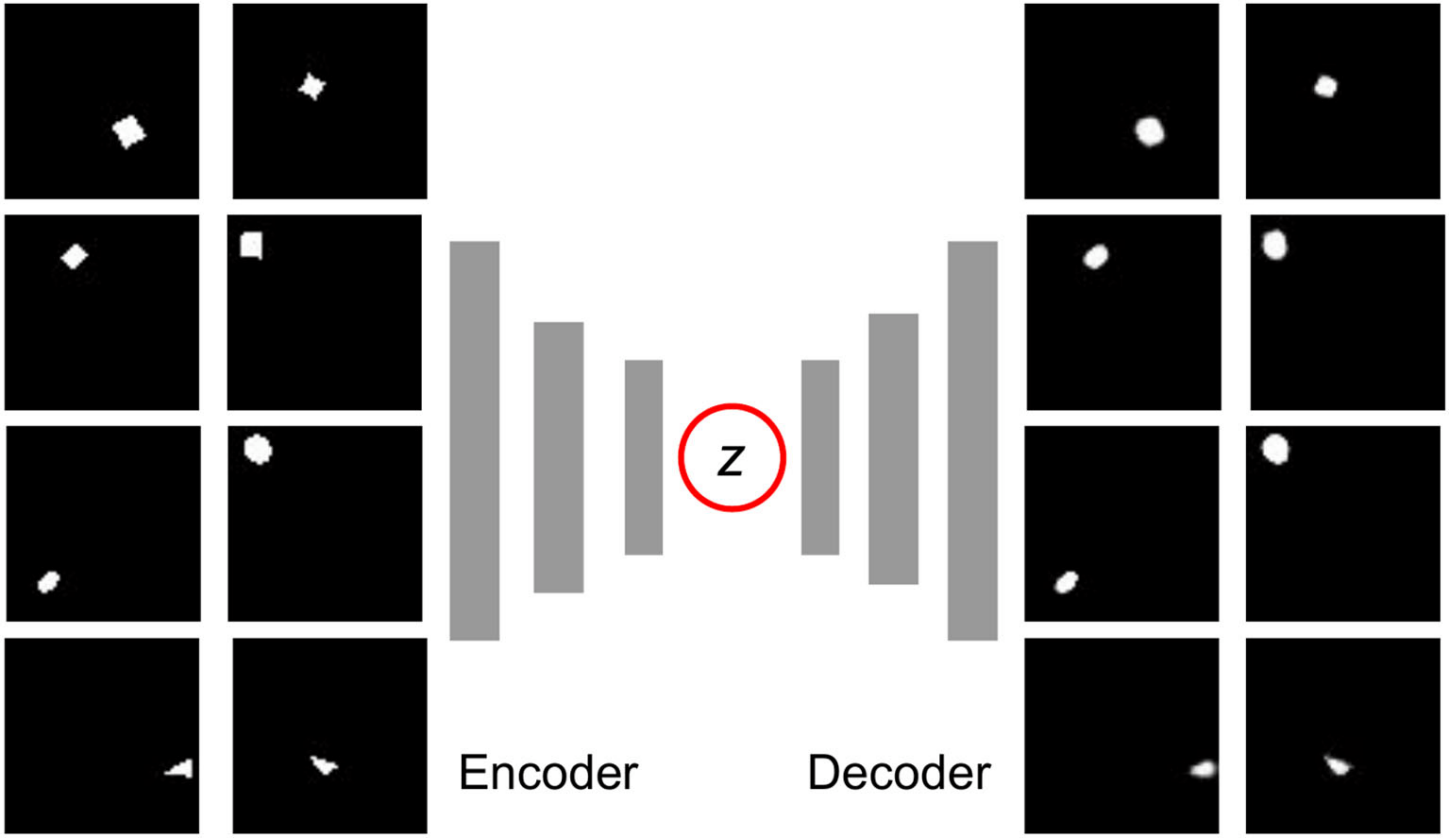
The training dataset (left) and reconstruction (right) of the Variation Autoencoder (VAE). All of the images are sized 64x64. The dataset includes 20,000 images, and all the object samples are illustrated in Figure 5. As we can see, the position and general shape are well reconstructed, even though some details such as the rotation angle and sharp corners are not exactly the same as the originals.

To deal with high-dimensional image inputs, we train a latent representation of the state by VAE. A VAE is a probabilistic generative model composed of an encoder that converts state *x* into a prior distribution *q*_θ_(*z*|*x*) and a decoder that converts the latent variable *z* back to a state distribution *p*_ω_(*x*|*z*) as is shown in Figure 4. Both the encoder and decoder are deep networks with trainable parameters (θ and ω). The model is trained by minimizing the reconstruction loss of original states *x* [the first term in Equation (2)] and forcing the latent representation *z* to be similar (in KL divergence Kullback and Leibler, 1951) to a prior distribution (second term) at the same time.


(2) $$\begin{array}{r} L\left(\theta ,\omega \right)=-E_{q_{\theta }\left(z|x\right)}\left[\log p_{\omega }\left(x|z\right)\right]+KL\left(q_{\theta }\left(z|x\right)||p\left(z\right)\right) \end{array}$$

We take Gaussian distribution as prior here, the mean and log-variance of the Gaussian distribution can be represented as follows:


(3) $$\begin{array}{r} q_{\theta }\left(z|x\right)=N\left(\mu_{\theta }\left(x\right),\sigma_{\theta }^{2}\left(x\right)\right) \end{array}$$

**Figure 5:**
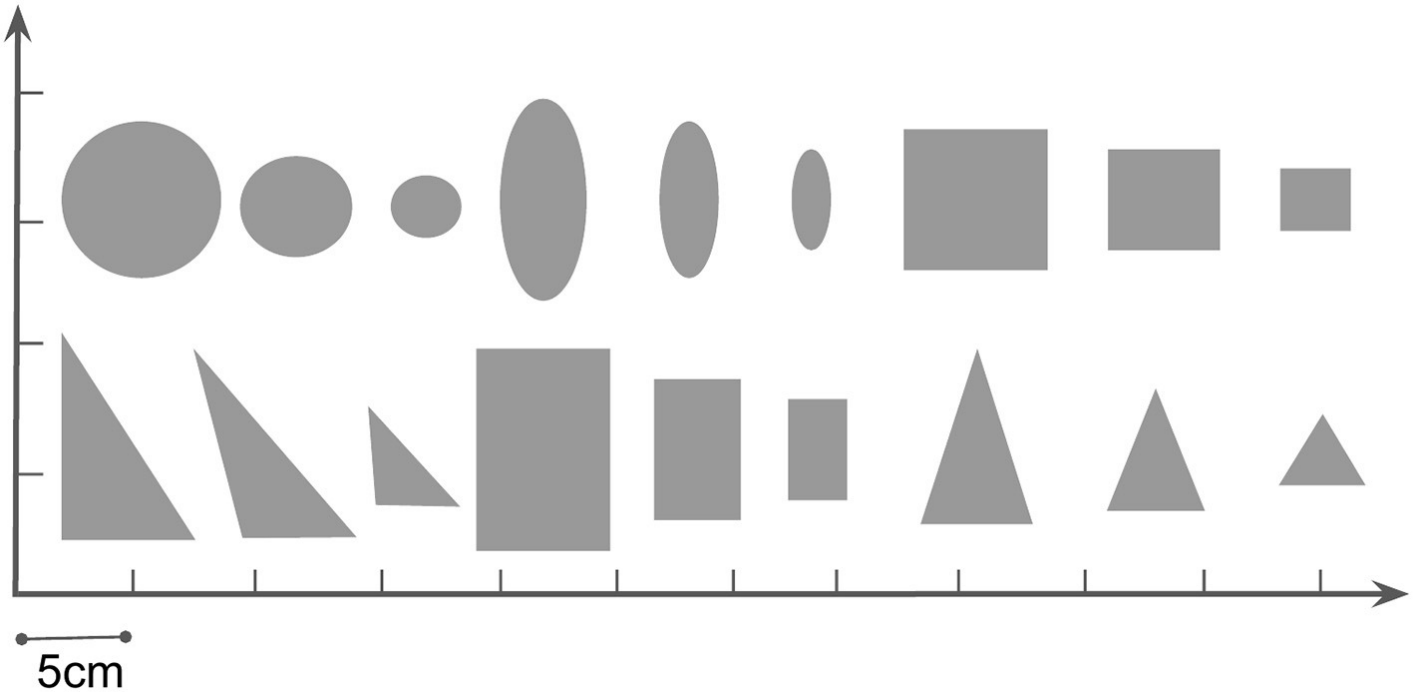
We use 18 different objects in our pushing task, all of them of 2 cm height. The physical parameters which can affect the dynamics are randomized from a range in Table 2 with automatic domain randomization (ADR) during training.

In our task, we only care about the pose and shape of the object and target in the image; color and texture information can be ignored. We filter the image from the bottom camera by color (red) and obtain the masks as in Figure 3. The VAE is trained on these one-channel 64x64 masks. We collect a mask dataset by randomizing the pose of different objects on the table. Figure 5 shows all the objects used in the dataset.

#### 2.2.3. Vision-Proprioception Model

One of the most challenging parts of visual RL tasks is that the agent needs to learn both perceptions from high dimensional data and the control policy simultaneously. Given an image, a usual perceptron like multi-layer perceptron (MLP) or CNN encodes all the information from the input. However, human beings only pay attention to related information, which is more efficient when solving a complicated task. Besides visual input, proprioception is also an essential channel among all the modalities. Inspired by the way human beings solve a task through both vision and proprioception, our vision-proprioception model fuses these two and performs planning in the fusion space.

We embed the object mask *m*_*o*_ and goal mask *m*_*g*_ into a latent space *z* with the pre-trained encoder *e* in Section 2.2.2, getting the latent object state *z*_*o*_ = *e*(*m*_*o*_) and latent goal state *z*_*g*_ = *e*(*m*_*g*_). As the latent variable *z* samples from Gaussian distribution $q_{\theta }\left(z|x\right)=N\left(\mu_{\theta }\left(x\right),\sigma_{\theta }^{2}\left(x\right)\right)$, we take the mean of the encoder μ_θ_(*x*) as the state encoding. With the planar position of the pusher *p*_*r*_ = [*x, y*] easily obtained from forward-kinematics on the joint-angles during robot manipulation, we construct a fusion state space $S=\left[p_{r},z_{o},z_{g}\right]$ in which the policy $\pi_{\phi }\left(a_{t}|s,s\in S\right)$ does its planning. The action *a* = [*a*_*x*_, *a*_*y*_] is a continuous vector, representing the pusher's 2-D velocity in the motion plane. We use squashed action implementation: *a* = tanh(ā), in which $ā\sim N\left(\mu_{\phi }\left(x\right),\sigma_{\phi }^{2}\left(x\right)\right)$ (Raffin et al., 2019).

### 2.3. RL With Vision-Proprioception Model

**Figure 6:**
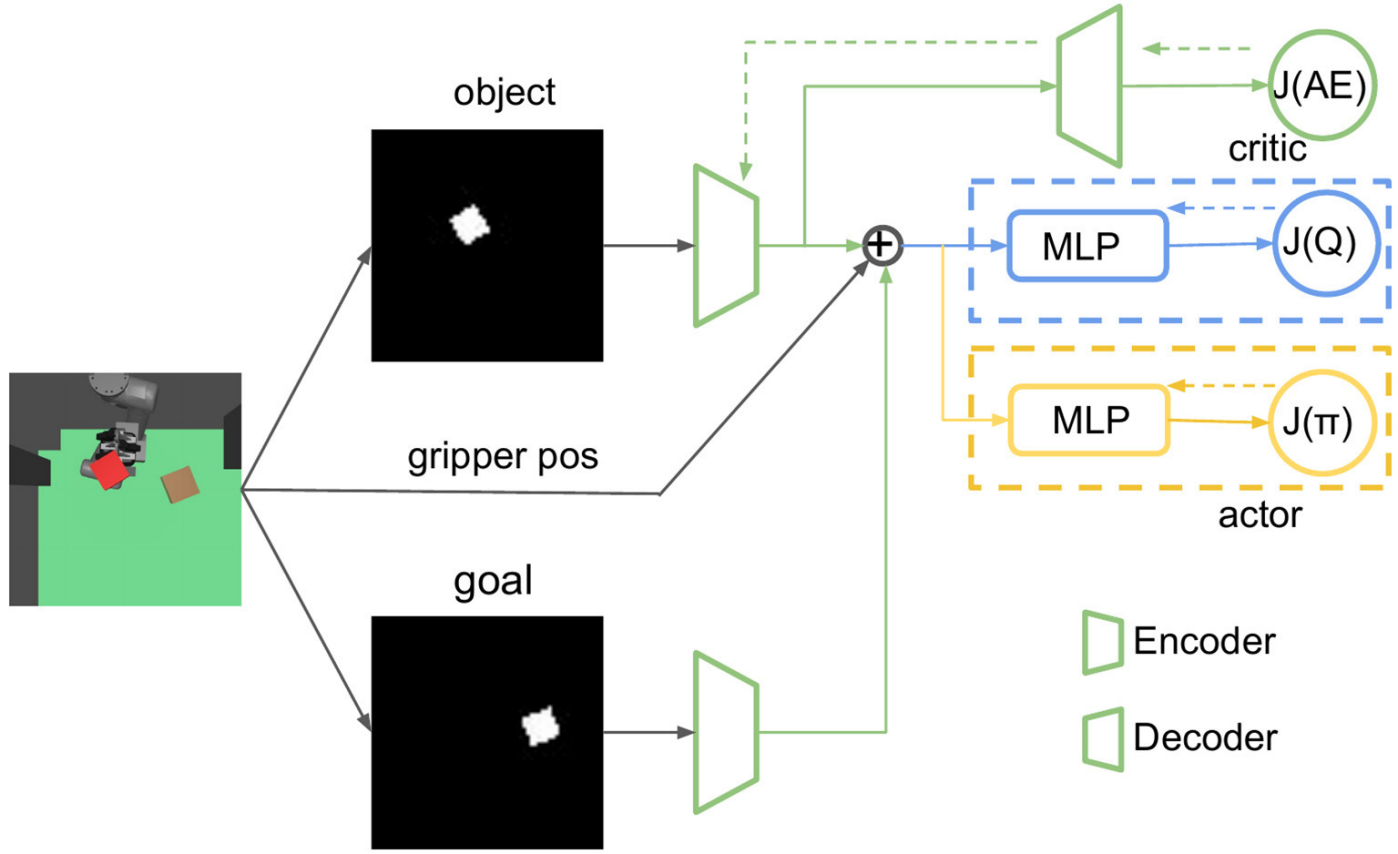
The network structure of our reinforcement learning (RL) framework, in which dashed arrows represent back propagation. VAE and RL are trained separately, encoders from VAE do not update during the RL training process. The critic and actor parts are optimized by value target *J*(*Q*) and expected future return *J*(π).

In this part, we show how to train RL with the model we propose in Section 2.2.3. To improve the sample efficiency of RL, we use the stochastic off-policy algorithm SAC (Haarnoja et al., 2018) with the goal relabelling trick HER (Andrychowicz et al., 2017). With an entropy regularization as part of the optimization, the policy is trained to maximize the expected return and the policy entropy, which is a randomness of the policy. The general training process is similar to other off-policy RL algorithms like DDPG and TD3 by optimizing two targets: value function *J*(*Q*) and policy function *J*(π). The model architecture is shown in Table 1. FC(), Conv(), and ConvT() represent the fully connected, convolutional, and transposed convolutional networks, respectively. The arguments of FC() and Conv() / ConvT() are [node] and [channels, kernel size, stride]. The training process of the whole framework is shown in Figure 6. VAE is pre-trained before being used in RL; the two encoders for object and goal share the same weights. During the experiment, we can understand what the agent sees by visualizing the reconstruction image from the decoder, which turns out to be quite useful for debugging purposes during the training process.

**Table 1:** Model architecture.

**Model**	**Architecture**
Encoder	Conv([[32,4,2],[64,4,2],[128,4,2],[256,4,2]])
	FC([256,6])
Decoder	FC([256,1024])
	ConvT([[32,5,2],[32,5,2],[16,6,2],[1,6,2]])
Actor	FC([128,256,64,2])
Critic	FC([128,256,64,1])

#### 2.3.1. State Space and Reward Specification

We use the following symbols in Algorithm 1: *p*_*r*_, *p*_*o*_, and *p*_*g*_ are the ground truth positions of the robot pusher, center of the object, and goal, respectively. Both *p*_*o*_ and *p*_*g*_ are only used to compute the step reward during the training process in simulation, but not during tests or in the real robot experiment. *x*_*o*_ and *x*_*g*_ are pixel observations of the object and goal, *z*_*o*_ and *z*_*g*_ are corresponding encodings. Different reward functions can lead to diverse training results. Previous work trained the robot in the real world and computed rewards in the latent space of the pixel observation (Xu et al., 2020). However, computing rewards from the ground truth data is also possible in simulation, as all ground truth information is available. In our work, we consider three different kinds of reward functions. The first is a dense reward in latent space:


(4) $$\begin{array}{r} r\left(z_{o},z_{g}\right)=-||z_{o}-z_{g}|| \end{array}$$

and the second is sparse reward computed by the ground truth state:


(5) $$r(p_{o},p_{g})=\{\begin{array}{ll} -1, & \text{if}\|p_{o}-p_{g}\|\;>threshold\\ 0, & \text{if}\|p_{o}-p_{g}\|\;⩽\;threshold \end{array}$$

**Algorithm 1:**
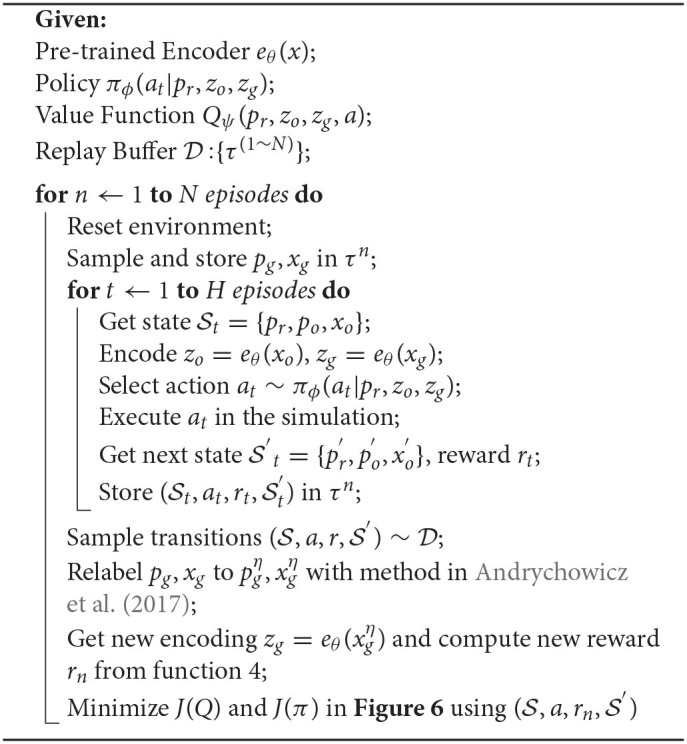
RL with Vision-Proprioception Model.

The third is dense reward with ground truth state:


(6) $$\begin{array}{r} r\left(p_{o},p_{g}\right)=-||p_{o}-p_{g}|| \end{array}$$

The results are compared in the next section.

#### 2.3.2. Sim2Real

We use Robogym (OpenAI, 2020) as the framework and build our simulation environment according to our UR5 platform. Domain randomization is used to bridge the gap from simulation to the real world. With enough variability, the object in the real world may be a variation from the randomized domains. We apply 18 differently shaped objects during the training process, as shown in Figure 5, and randomize their physical parameters, including mass, sliding/rotation friction coefficient of the object from a reasonable range. Details of the physical parameters are shown in Table 2. We randomize 20 different combinations of physical parameters for each of the objects. At the beginning of each episode, one combination of the physical parameter is chosen and remains unchanged during the episode (50 action steps in the training process). During training and testing, we find an apparent gap between simulation and the real world in all objects' rotational motion: given the same pushing action, the object shows more rotation in the real world.

**Table 2:** Dynamic parameters and their ranges in simulation.

**Parameters**	**Range**
Size	[4, 15] cm in length, fixed height=2cm
Mass	[0.05, 0.3] kg
Sliding friction coefficient	[0.1, 1]
Rotation friction coefficient	[0.001, 0.01]
Damping coefficient	[0.01, 0.015]

## 3. Results

We first test our algorithm by training an agent in simulation, then evaluate the training results by applying the model directly to a real robot platform. We build the same hardware setup for simulation and the real robot as shown in Figure 3. The red rectangle represents the object to be pushed, and blue is the goal position. In a simulation (Todorov et al., 2012), the non-collision goal object can be rendered conveniently. However, in real experiments, for each episode, we first put the object at the point we want, take an image with the bottom camera and record it as the target image. Under human interference, the robot can consistently switch the pushing side and keep pushing the object to the target. We use the same online controller (Ruppel et al., 2018) to translate Cartesian motions of the pusher into joint-space robot commands as in our previous pushing research (Cong et al., 2020).

### 3.1. Simulation Results

**Figure 7:**
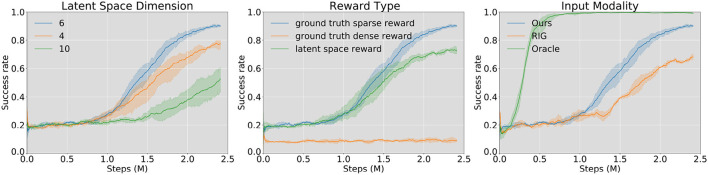
Learning results in simulation, success rate vs. training steps. From left to right: model trained with different VAE latent space dimensions (left), reward functions (middle), and input modalities (right). Through experiments, we find that the best performance comes from the model with latent space dimension *d*_*z*_ = 6, trained with the ground truth sparse reward function. “Oracle” in the right figure is the training results of a state-based agent on one single object (cylinder, *d* = 4*cm*). “RIG” (Nair et al., 2018) takes only the image as input. We set random explorations at the first 200 episodes for each training process, leading to an initial success rate of around 20% for each learning curve.

We evaluate our method against two prior model-free SOTA algorithms and do ablation studies to determine how critical each component of our method is. As is shown in Figure 7, we compare the learning performances with the pushing success rate. One episode is considered a success if the final center point distance is within the threshold (5 cm) we set in Equation (5). Orientation error is not considered in the reward function. To our best knowledge, RL with imagined goals (RIG) is the SOTA algorithm for the visual pushing task (Nair et al., 2018, 2020). We choose RIG as the baseline method. Besides, we also give the results with direct access to state information, including the robot's end-effector position and the object's pose (Oracle). However, because the interaction dynamics of differently shaped objects differ significantly, one state-based policy can neither learn to push all 18 candidates used in our experiment nor train on one specific object and then generalize to another object. Therefore, the “Oracle” learning curve is the learning result of pushing **one specific cylinder**. For the other experiments, **one random object** is selected from all the candidates at the beginning of each episode.

During experiments, we find that two components have the most significant influence on the training performance: (1) the dimension of VAE latent space and (2) the reward function type. We first compare learning results using VAE models with different latent space dimensions (Figure 7, left). During all training processes of the VAE, as the training epochs keep rising, the model goes from under-fitting to over-fitting. Most of the over-fitting happens between the range of (60, 90) epochs. For the models with different latent spaces, we choose the saved ones, which occur just before the over-fitting happens. Through analysis, we find that the best latent dimension is 6. This may be because it is a suitable dimension to remember the shape and pose of the object, and at the same time, its representation is not too complicated for the agent to learn an effective policy. Lower latent space dimension (4) is not enough to represent all necessary features of the mask image. However, a higher dimension (10) also increases the learning difficulty of the agent.

One of the differences between our work and Nair et al. (2018) is that we train our model in simulation and use it in on a real robot, which reduces training time considerably and facilitates access to the environment ground truth state, while the above authors directly train their agent in the real world. We apply three different reward functions (Section 2.3.1) in the training time and find that (1) sparse reward from ground truth state (Equation (5)) leads to the best pushing performance (90% success rate), (2) dense reward in latent space (Equation (4)) can also guide the agent to a working policy, (3) dense reward from ground truth state (Equation (6)) is invalid as is shown in Figure 7.

### 3.2. Real Robot Verification

**Figure 8:**
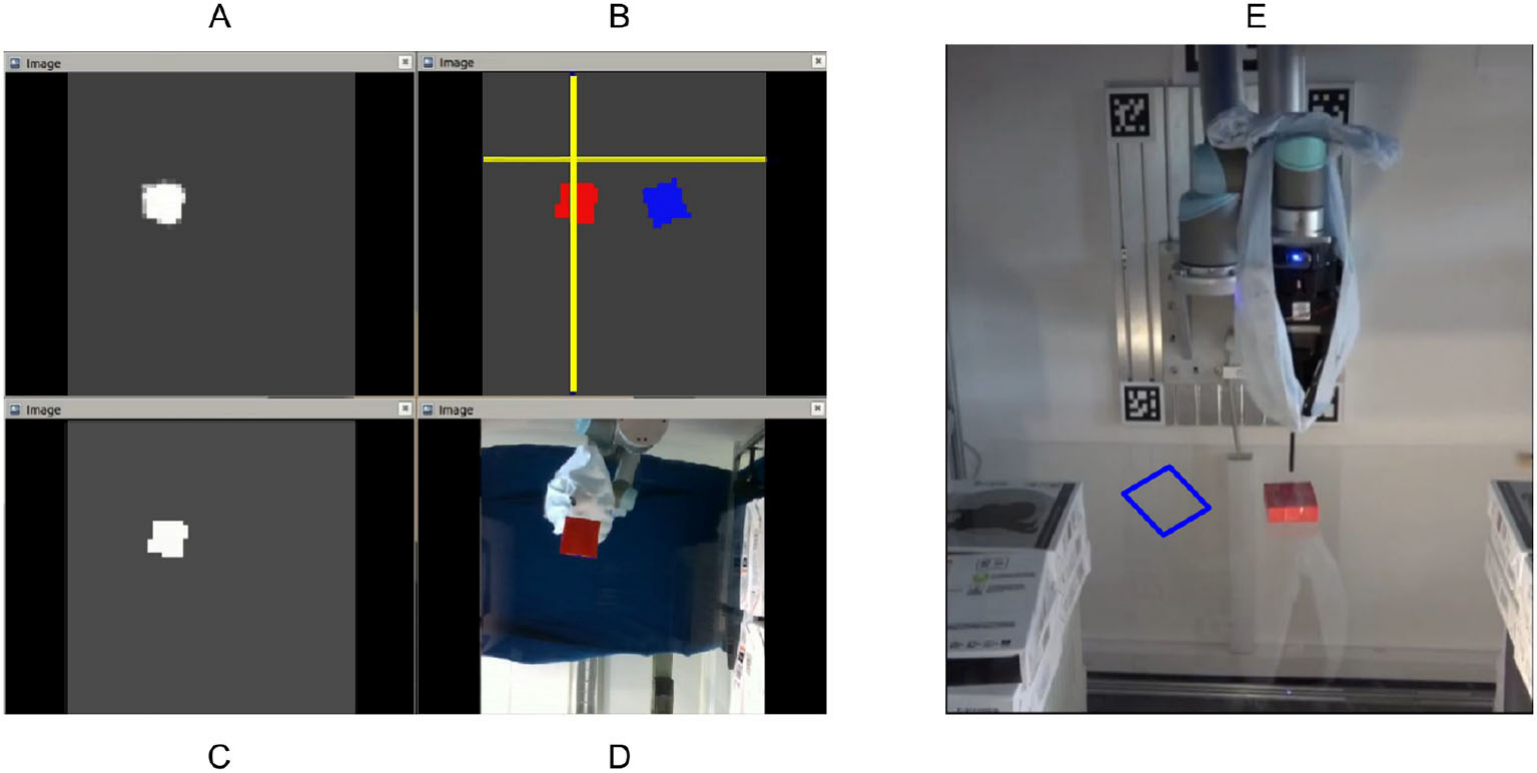
The layout of the visualization tool in the experiment. A comprehensive view is given in **(B)**: the blue and red rectangles represent the target and real-time object pose, respectively, the yellow cross represents the pusher position. **(A,C)** are the real-time object masks after the color filter and the mask reconstruction from the decoder. We find it quite useful to visualize the two masks during debugging for the experiment. **(D)** shows what the robot sees from the bottom camera and **(E)** is the front view.

**Figure 9:**
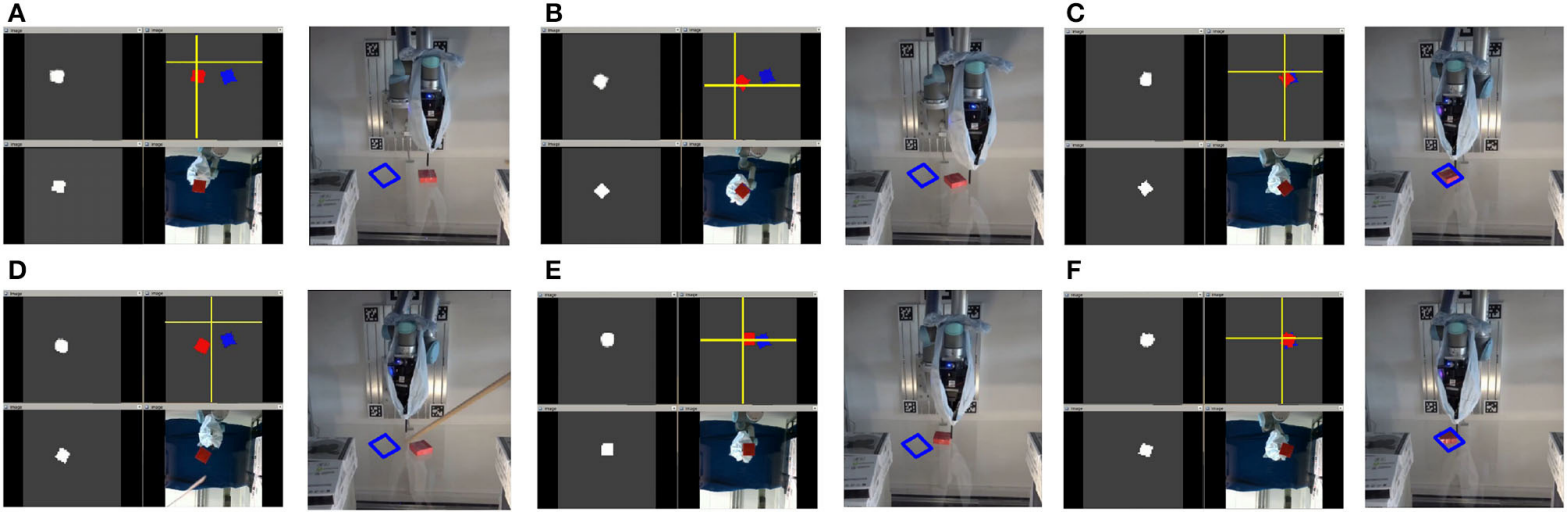
A cuboid pushing process under human interference on the real robot platform. The object is put at an initial pose (marked with a blue box), the bottom camera takes an image and records it as the target image, which is the blue mask. The robot keeps pushing the object to the target during the whole episode. From **(A–C)**, we can also see that the robot is switching the pushing side as in the simulation. In **(C)**, the first pushing target is reached, and we give an active interference in **(D)**. From **(D)** to **(F)**, the robot adjusts the pusher consistently and finishes the second pushing process successfully.

This part evaluates whether our model can be transferred to the real world and manipulate unseen objects (with similar shape and size) without any fine-tuning. All the models in our method, including the VAE and policy network, are trained in simulation. The visualization tool and the real pushing experiment are shown in Figures 8, 9. We visualize both the original object mask and its reconstruction in real-time to check whether the information in latent space is correct or not.

**Figure 10:**
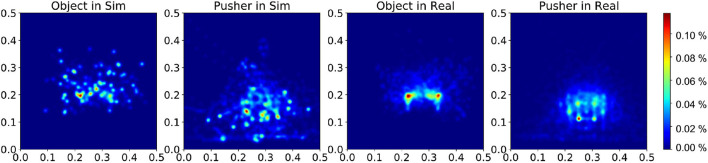
Distribution of the object and pusher's position during tests in simulation and real experiments. The color bar represents the occurrence frequency.

**Figure 11:**
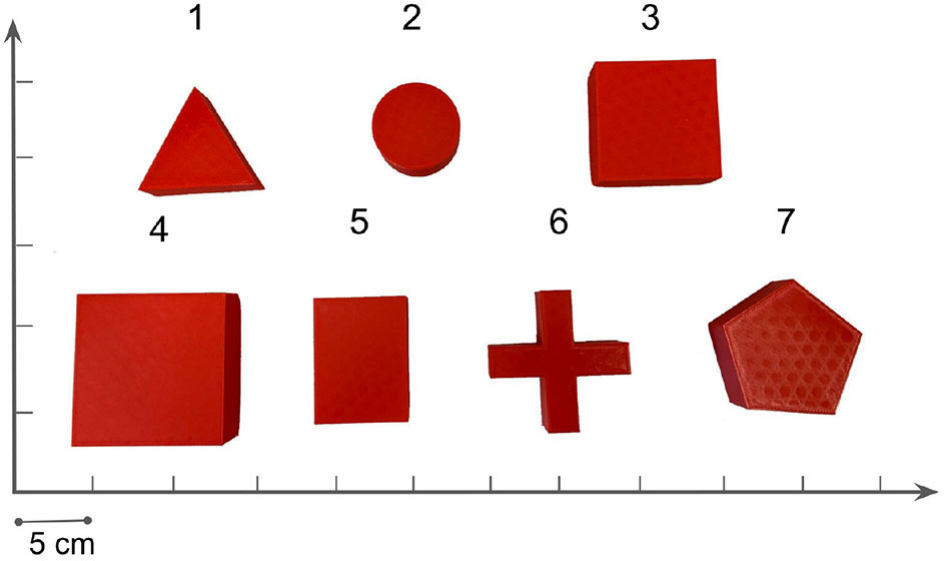
Illustration of 7 tested objects in the real experiments. Object numbered from 1–3 are training objects in the simulation. Object 4 and 5 have similar shapes but different size with the training objects, but object 6 and 7 are novel in shape.

During experiments, we find that the interaction dynamics in the real world are different from that in simulation, especially on the rotation motion of the object. The objects turn easily around the vertical axis in the real world under the robot's pushing actions. Even though we randomize the physical parameters in simulation from a wide range, the gap cannot be eliminated. We assume this is because the simulator simplifies the contact model. The position distribution frequency from 500 episode trajectories is shown in Figure 10. We set the same robot working space and goal randomization space for simulation and the real world. However, because the objects rotate easily in the real world, the robot needs more adjusting motions to push the object to goal positions than simulation. Most of these unexpected adjusting motions happen around the workspace center, rendering the object and pusher trajectory distribution more intensive in the center part. To analyze the transfer performance of our model, we measure the distance between the goal position of the object and the final position (without orientation), also the corresponding time consumption. We test 3 objects (1–3 in Figure 11) from the training dataset and four unseen objects (4–7 in Figure 11) in the experiment. The results in Table 3 show that our method shows robustness to unseen objects. More adjusting pushing actions also mean more time consumption for each push. Object 7 (the pentagon) has a novel shape which is not included in the training object, but our model can deal with it successfully. Except for the object 6, the mean distance between goal and final position is within 5 cm in both simulation and the real world. We analyzed that the concave shape of object 6 is not learned by our Encoder. Therefore, the latent state only extracts the position information from the mask, but the shape feature is not well represented.

**Table 3:** Comparison of pushing results.

**Object number**	**In simulation**	**In real world**
1	3.2 (cm) / 5.5 (s)	3.5 (cm) / 22.9 (s)
2	3.5 (cm) / 4.6 (s)	3.7 (cm) / 18.6 (s)
3	2.9 (cm) / 4.3 (s)	3.1 (cm) / 19.4 (s)
4	3.6 (cm) / 5.6 (s)	3.9 (cm) / 21.5 (s)
5	4.2 (cm) / 6.2 (s)	4.5 (cm) / 23.3 (s)
6	5.2 (cm) / 8.2 (s)	5.5 (cm) / 27.7 (s)
7	3.8 (cm) / 4.4 (s)	3.8 (cm) / 20.3 (s)

## 4. Discussion

In this study, we present a self-supervised pixel-based method that can encode visual inputs into latent space and fuse with a robot's proprioception into one model, to solve the task of planar pushing and achieve a competitive advantage over a state-based method. (The latter method can only be trained on a single object without the ability to generalize to other objects.) Our model is trained in a simulation environment and can be transferred to the real world without fine-tuning. Real experiment results show that the model is of high robustness to similar but unseen objects.

The core idea of our method is to force the agent only to pay attention to useful information in the image and fuse the encoding with information from other perceptions in the environment, making use of all task-relevant inputs from multiple channels. A vision-proprioception model is proposed as the controller and trained with SAC. We believe our method can be taken as an inspiration to extract useful information from different modalities and fuse them for end-to-end decision-making problems, improving learning efficiency and performance in real robot RL tasks.

Based on current research, our future work will be adding a top-down attention mechanism into RL tasks. One limitation of our current method is that the agent cannot intelligently judge whether the information is helpful in the task. The ability to learn and infer task-relevant information from sequential observations could solve more complicated tasks and make our algorithm even more generalizable.

## Data Availability Statement

The original contributions presented in the study are included in the article/Supplementary Material, further inquiries can be directed to the corresponding author/s.

## Author Contributions

LC designed the algorithm and experiments. HL and NH helped with the robot setup. PR provided the robot online controller. All the authors contributed to valuable discussions in this paper.

## Funding

This research was funded by the German Research Foundation (DFG) and the National Science Foundation of China (NSFC) in project Crossmodal Learning, DFG TRR-169/NSFC 61621136008. LC was supported by the China Scholarship Council (CSC).

## Conflict of Interest

The authors declare that the research was conducted in the absence of any commercial or financial relationships that could be construed as a potential conflict of interest.

## Publisher's Note

All claims expressed in this article are solely those of the authors and do not necessarily represent those of their affiliated organizations, or those of the publisher, the editors and the reviewers. Any product that may be evaluated in this article, or claim that may be made by its manufacturer, is not guaranteed or endorsed by the publisher.
